# The importance of evaluating standard monitoring methods: Observer bias and detection probabilities for moose pellet group surveys

**DOI:** 10.1371/journal.pone.0268710

**Published:** 2022-07-27

**Authors:** Anne Loosen, Olivier Devineau, Barbara Zimmermann, Karen Marie Mathisen

**Affiliations:** Department of Forestry and Wildlife Management- Evenstad, Inland Norway University of Applied Sciences, Elverum, Norway; Universitat Autonoma de Barcelona, SPAIN

## Abstract

Counting is not always a simple exercise. Specimens can be misidentified or not detected when they are present, giving rise to unidentified sources of error. Deer pellet group counts are a common method to monitor abundance, density, and population trend. Yet, detection errors and observer bias could introduce error into sometimes very large (spatially, temporally) datasets. For example, in Scandinavia, moose (*Alces alces*) pellet group counts are conducted by volunteer hunters and students, but it is unknown how much uncertainty observer error introduces into these datasets. Our objectives were to 1) estimate the detection probability of moose pellet groups; 2) identify the primary variables leading to detection errors including prior observer experience; and 3) compare density estimates using single and double observer counts. We selected a subset of single observer plots from a long-term monitoring project to be conducted as dependent double observer surveys, where primary and secondary observers worked simultaneously in the field. We did this to quantify detection errors for moose pellet groups, which were previously unknown in Scandinavia, and to identify covariates which introduced variation into our estimates. Our study area was in the boreal forests of southern Norway where we had a nested grid of 100-m^2^ plots that we surveyed each spring. Our observers were primarily inexperienced. We found that when pellet groups were detected by the primary observer, the secondary observer saw additional pellet groups 42% of the time. We found search time was the primary covariate influencing detection. We also found density estimates from double observer counts were 1.4 times higher than single observer counts, for the same plots. This density underestimation from single observer surveys could have consequences to managers, who sometimes use pellet counts to set harvest quotas. We recommend specific steps to improve future moose pellet counts.

## Introduction

Sign surveys have a long tradition in the field of ecology, such as counting the number of birds seen from a point location, the number of frogs heard during a set time interval, or the number of carnivore scats seen along a transect line. The number of specimens observed can be used as an index of abundance, density, or population trend. However, counting is perhaps not as simple as it seems [1]. For example, Prater [2] found that observers counting birds from photographs (i.e., true values known) consistently overestimated the number of individuals when counting small groups of birds, but underestimated their numbers when counting large groups. Errors associated with sign surveys, such as observation (e.g., species misidentification) or detection error (e.g., animal was present but went undetected) lead to biased estimates of occupancy, density, or abundance [3–5]. Recently, statistical advances have attempted to account for these errors [6, 7]. For example, the advent of methods to account for differences in detection and movement skyrocketed with the use of camera traps [3]. Yet, certain sign survey methods lag in widespread methodological advancements and acceptance.

A common sign survey for monitoring deer (Cervidae) is counting their feces (i.e., pellet group counts) as a proxy for animal distribution, abundance, population trend and assessing browsing intensity [8–11]. The primary issue with counting pellet groups, as with any sampling method, is identifying the sources of error [8, 12, 13]. For example, pellets may be removed or made less visible through heavy rain events or trampling [14, 15], or decompose quickly if the habitat is damp or if there is insect activity [16]. Additionally, if pellet groups are present, there is a possibility they may not be detected (i.e., the detection probability). Detection probabilities of deer pellet groups can vary widely, especially based on pellet size, environmental conditions, weather, and observer experience. In a study of Columbian black-tailed deer (*Odocoileus hemionus*) and Roosevelt elk (*Cervus elaphus roosevelti*) in a temperate coniferous rainforest, pellet detection probabilities (defined as ‘*p*’) ranged from < 0.2 to 1.0, depending upon observer and distance of the pellet group from the transect line [17]. In another study, pellet groups from the muntjac (*Muntiacus muntjac*), a small deer resident to tropical forests of India, had very low detection probabilities of *p =* 0.03 (SE = 0.02; [18]), meaning they were almost impossible to detect when present. Pellet group counts (hereafter, pellet counts) continue to be used extensively because they do not require expensive equipment, technicians can be trained quickly, and surveys can be done across large areas. However, methods to quantify and account for sampling and detection errors are not widely implemented.

Moose (*Alces alces*) are the largest member of the deer family with comparatively large fecal pellets (2–3.5 cm; [19]). Moose pellet groups are typically identified by a single observer, and surveys are often repeated annually or semi-annually [20–23]. However, habitat heterogeneity, environmental conditions, and observer skill level introduce variation in the accuracy and precision of pellet counts [19, 24, 25]. For example, immediately after snow melt and before spring green-up, moose pellets are highly visible [24]. However, early green-up, late-season snow, or inexperienced observers can lead to imperfect detections [19, 24, 26].

Despite the widespread use of moose pellet counts as a direct index of moose density, abundance, or population trend, few studies incorporate detection error because it is largely assumed to be negligible since moose pellets are so large. In Scandinavia, for example, moose density (*D*) is often calculated as the number of observed pellet groups divided by the average defecation rate of 14 pellet groups per day for an accumulation period [21, 27–30]. This formulation, however, assumes perfect detection and minimal pellet decay during the winter period, which could lead to underestimates of moose abundance if these assumptions are incorrect. For a heavily hunted species such as moose, where between 18–35% of the population are harvested each autumn in Norway [31, 32], any systematic bias in population estimates could lead to a mismatch between population goals and management strategies.

Pellet counts are conducted by volunteer hunters in Sweden to track moose density and population trends, and by research projects in Scandinavia. Thus, pellet count datasets are formed from volunteer contributions, which we loosely term ‘citizen scientists’. Citizen science, the involvement of citizens in scientific research and knowledge production [33], allows researchers and managers to collect data across spatio-temporal domains that would otherwise be too costly to collect [33–35]. This is a growing field because of the availability of ‘free’ labor. Yet, these data have trade-offs such as observer bias as a result of (in)experience, the ease of implementing the sampling regime, and the spatial bias of data (i.e., clustering of data around urban areas; [36]). There is new emphasis on validating the quality of citizen science data [33, 37] but this step is not universally applied and not enough is done to quantify potential biases.

We designed a study using single and double observer survey methods to count moose pellet groups in southern Norway. Our objectives were to 1) estimate the detection probability of moose pellet groups; 2) identify the primary variables leading to detection errors including prior observer experience; and 3) compare density estimates using single and double observer counts. We predicted higher detection probabilities with double observer compared to single observer surveys, with more search time, for more experienced observers, and higher density estimates with double observer counts.

### Study area

**Fig 1:**
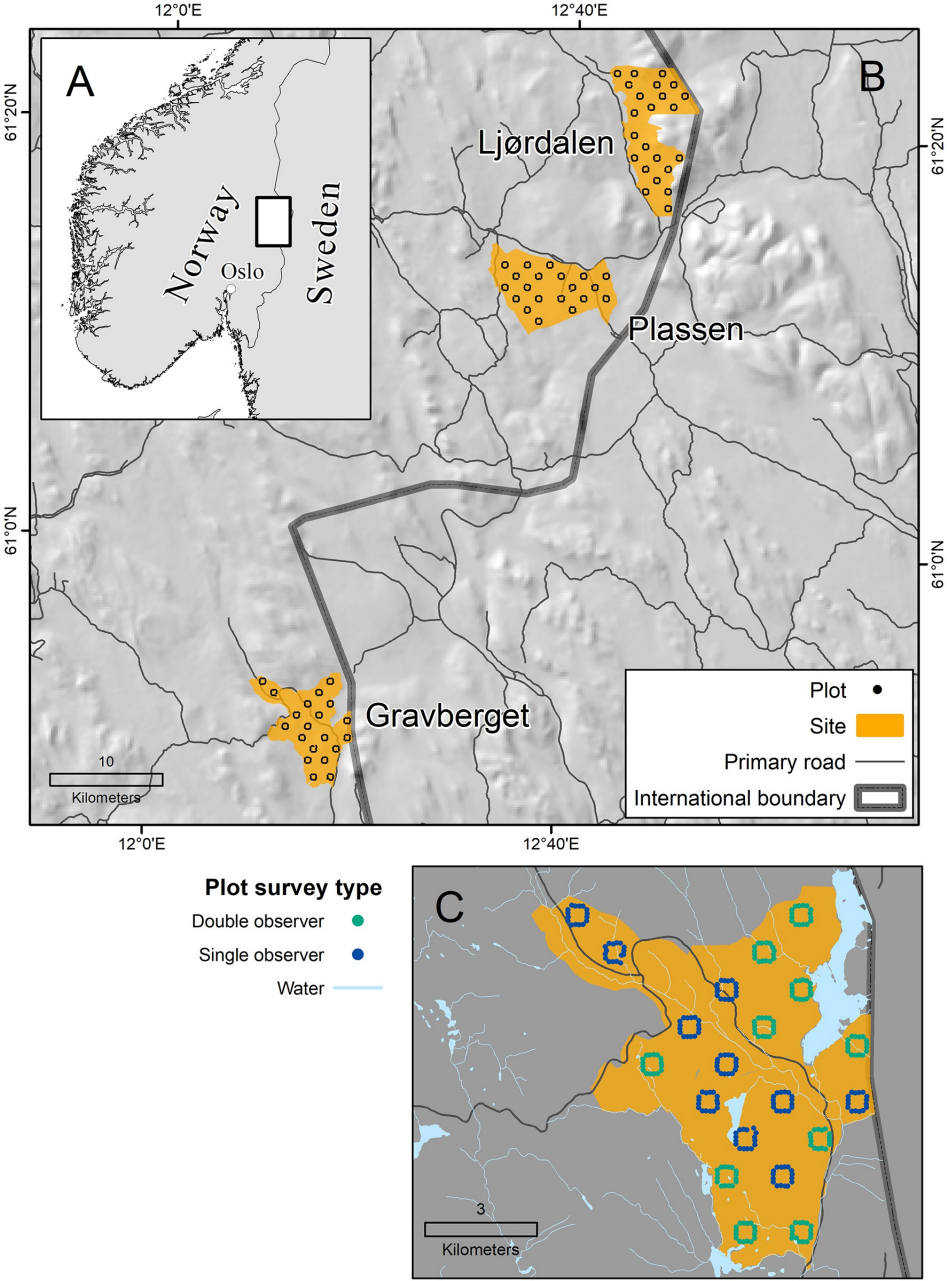
Maps of A) study area in southern Norway; B) each of the three sites (Gravberget, Plassen, Ljørdalen) which contained 20–21 quadrats. Background map is a 25-m digital elevation map; C) each quadrat contained 16 plots. A subset of long-term monitoring plots was completed as double observer surveys. Gravberget is shown as an example. Digital elevation maps were reprinted from European Digital Elevation Model (EU-DEM, version 1.1) under a CC BY license, with permission from Copernicus Sentinel Data and Service Information, original copyright 2016.

**Fig 2:**
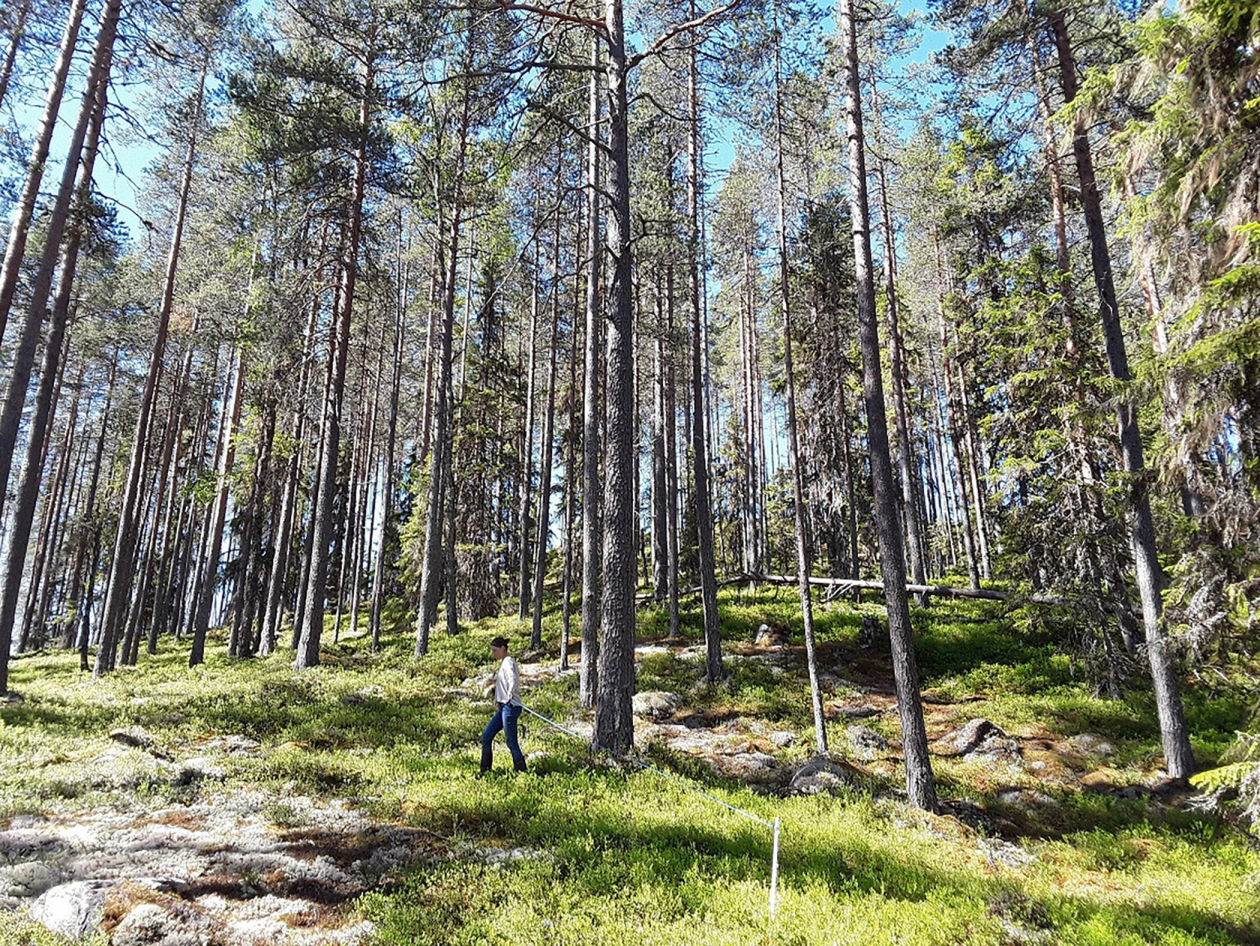
An observer conducts a moose pellet group survey in Norway. This plot represents a typical Norwegian production forest with minimal structural diversity. Photo credit Annie Loosen.

Our study area lies between 60.8° and 61.4° N and 12.2°–12.7° E in Innlandet County in southern Norway (Fig 1). Elevation ranges from 265–750 m above sea level. The area experiences cold (mean January temperature 2011–2018: -9.3 C°) and snowy (mean winter snow depth 2011–2018: 39.0 cm; Norwegian Meteorological Institute) winters and short, cool summers. Land cover is dominated by boreal forests [38], which are managed for timber and pulp production based on even-aged forest management. Production forests, which are largely coniferous, typically undergo one pre-commercial thinning at 10–20 years to remove competing deciduous shrubs and trees (Fig 2). Stands undergo 1–2 thinning events at 40–50 years and 70–80 years to optimize commercial tree density. Moose prefer young forests for the high food availability [39].

Common tree species include Scots pine (*Pinus sylvestris*), silver birch (*Betula pendula*), downy birch (*Betula pubescens*), Norway spruce (*Picea abies*), grey alder (*Alnus incana*), rowan (*Sorbus aucuparia*), goat willow (*Salix caprea*), and aspen (*Populus tremula*). The shrub layer is dominated by bilberry (*Vaccinium myrtillus*), heather (*Calluna vulgaris*), and other dwarf shrubs (*Ericaceae* spp.). In boggy areas, *Sphagnum* spp. mosses are dominant [40]. In winter, moose typically migrate from summer ranges in higher elevation areas to low-elevation valley bottoms where snow depths are reduced [41–43]. Moose are the dominant deer species. Roe deer (*Capreolus capreolus*) and red deer (*Cervus elaphus*) are present in the area but occur at very low densities.

## Methods

### Pellet counts

**Fig 3:**
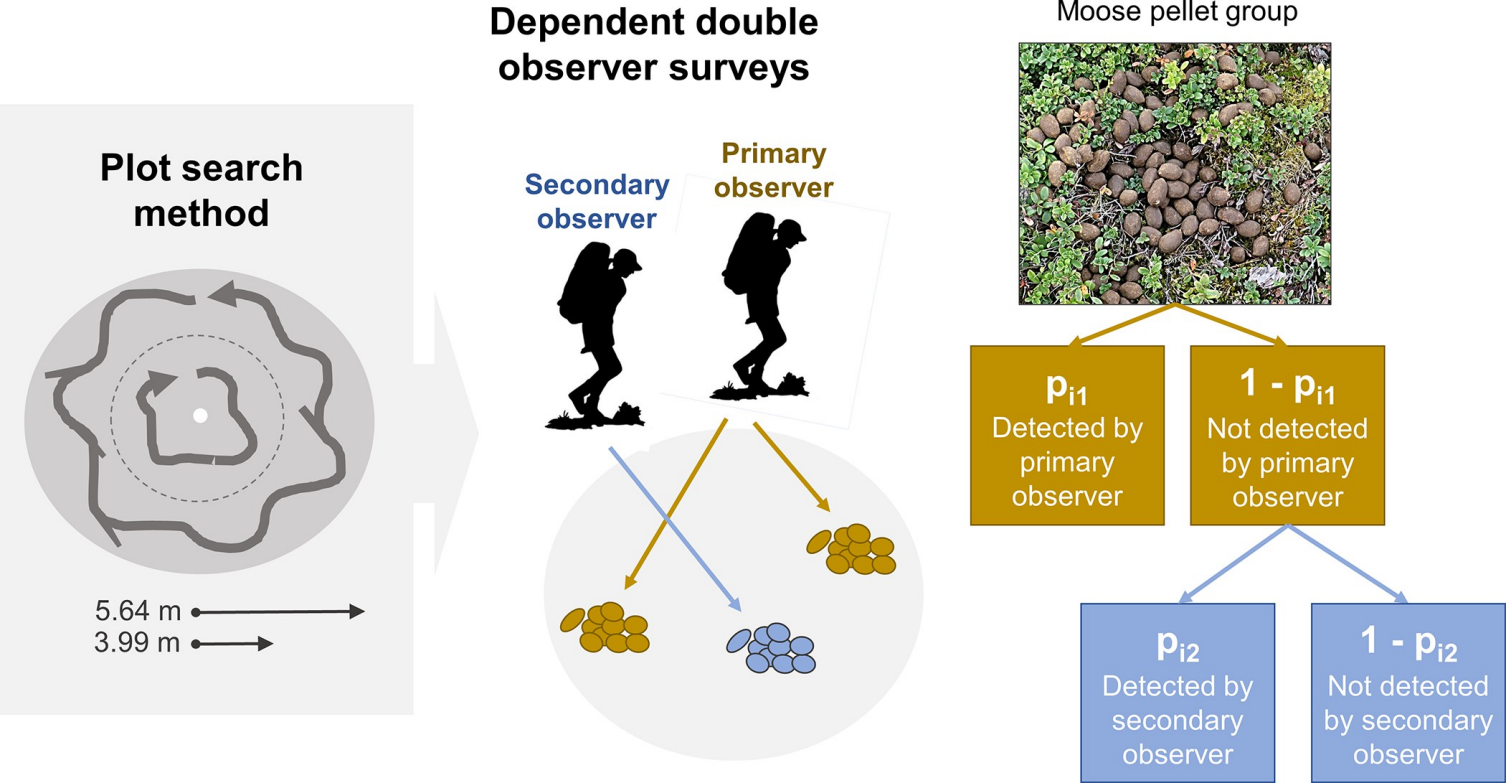
In the grey box at left, a diagram showing how each 100-m2 circular plot was searched. Observers first walked the ‘inner circle’ (the 3.99-m radius, indicated by the dotted grey line) in a zig-zag fashion to identify pellet groups. Once the inner circle was surveyed, the observer moved to the outer circle (5.64-m radius) and surveyed in the opposite direction. Each plot center was marked with a white pole. At center, a diagram showing the data collected during dependent double observer surveys. The primary observer (in brown) communicated the pellet groups seen to the secondary observer, who recorded the observations (here, two pellet groups). The secondary observer (in blue) recorded additional pellet groups the primary observer missed (here, one pellet group). At right, a diagram showing the three probabilities (p) estimated with dependent double observer surveys. Figure adapted from Powell and Gale 2015. Photo credit: Roos Ahlers.

As part of a long-term monitoring project [44, 45], we have plots (n = 992) nested within quadrats at three sites, which were named Gravberget, Plassen, and Ljørdalen. Sites were on average 30 km apart (SD = 17.8). Within each of the three sites, we systematically placed 20–21 quadrats of 500 x 500 m at a minimum distance between quadrats of 1 km. Along each quadrat border, we placed four circular plots every 100 m, resulting in 16 plots per quadrat (Fig 1. We counted pellet groups at individual plots. All plot centers were marked with a pole (Fig 2). Observers used a rope, which was marked at 5.64 m and 3.99 m, to measure the plot radii. Observers walked in the inner circle (radius = 3.99 m), zig-zagging within the circle to ensure the area was fully surveyed. Once the smaller circle was surveyed, observers moved to the outer circle (radius = 5.64 m) and searched in the opposite direction to ensure pellet groups obscured by vegetation could be seen (Fig 3). We counted deer pellet groups in late spring, shortly after snow melt (May or early June). We identified deer species according to morphological characteristics of the pellets [19]. Roe and red deer pellets are smaller and easily differentiated from moose pellets [19]. To count a pellet group, >50% of the pellets had to fall within the plot. Only piles with ≥20 pellets for moose and red deer and ≥10 pellets for roe deer were counted. We visually distinguished between fresh (current winter) and old (prior to winter) pellets. Winter pellets were typically brown, in pellet form, and positioned on top of leaf litter and forest debris, while summer pellets were often in patty form, covered by leaf litter, or had mold or fungus growth [21]. We included only winter pellets in this analysis. Pellets were removed from the plot each spring to avoid double counting the following year. One full day each field season (i.e., year), we trained observers in the field. Observers then worked independently for the duration of the field season. Most observers were students enrolled in an educational program at the authors’ institution and generally had little prior field research experience (Table 1).

**Table 1:** Table of plot- and observer-level covariates used in modeling. Covariates in bold were extrapolated from the 2018 dataset.

Covariate	Description	Plot-level	Observer-level
Weather	Weather conditions: foggy; rainy; cloudy (≥50%); partly cloudy (<50%); sunny/clear	X	
Snow cover	Percent cover of snow or standing water in the plot (0–100%). Visually estimated.	X	
Residual cover	Percent cover of forestry residues (i.e., branches) in the plot (0–100%). Visually estimated.	X	
Time of day	Time of day survey was started	X	
Search time	Time spent searching for pellet groups in the plot	X	
Julian day	Julian day of the year sampling occurred	X	
Forestry actions	Forestry activity: clearcut; thinned; scarified; clearcut and scarified; none of the above	X	
**Field layer**	Dominant field layer:	X	
	dwarf shrub; grass; fen; bog; lichen; small fern		
**Tree density**	Number of trees ≥ 0.3 m tall	X	
**Dominant tree**	Dominant tree species in the forest canopy	X	
**Cutting class**	Forest maturity index:	X	
	0: forestry not present (i.e., barren rock or bog);		
	1: clear cut; no regeneration;		
	2: visible regeneration, tree height < 10 m;		
	3: tree height > 10 m;		
	4: forest mature for logging, age of tree 55–75 years depending on productivity;		
	5: old growth forest		
Observer experience	Prior general field research experience:		X
	no experience;		
	1–6 months;		
	>7 months		
Pellet counting experience	Prior pellet counting experience:		X
	no experience;		
	1–6 months;		
	>7 months		

### Double observer surveys

To estimate detection error, we counted pellet groups using a dependent double observer survey method [12, 46]. Dependent double surveys have higher precision and are more time efficient in the field than independent double observer methods [13]. The primary observer surveyed the plot, pointing to and verbalizing observed pellet groups (Fig 3). The secondary observer recorded observations made by the primary observer while simultaneously searching for and recording additional pellet groups that the primary observer did not detect (Fig 3). Thus, the secondary observer counts were ‘dependent’ on the primary observer counts. All pellet group locations were drawn by hand on a data sheet in the field to ensure double counting did not occur (S1 Fig in S1 File). Observers switched primary and secondary observer roles between each plot [12, 17, 46]. Surveys thus resulted in two counts per plot: one count for the pellet groups seen by the primary observer and a second count for the additional pellet groups seen by the secondary observer. This method did not require observers to match or reconcile individual observations. Observers did not discuss their results. Double observers switched partners every day for the duration of the field season to increase knowledge mixing between all observers.

We aimed to complete 30% (n = 297) of the plots each year as dependent double observer surveys, based on previous years’ time effort and available observers. We used a random number selector to select 30% of the quadrats. We selected entire quadrats rather than single plots to minimize travel time between plots (i.e., a pair of observers could travel together to quadrats rather than meeting up after single observer surveys).

### Covariates

We recorded covariates which could increase variation in detectability (Table 1). At each plot, we recorded weather conditions, time of day, Julian day, forestry actions, and visually estimated the percent cover of snow or standing water and the percent cover of forestry residues (i.e., branches; see Table 1). We included also the total time spent searching for pellet groups in the plot. Because searching by primary and secondary observers occurred simultaneously, we recorded a cumulative search time (in minutes). There was no search time cut-off. The observer-level covariates we included in our models were ‘observer experience’, an index of prior field research experience, and ‘prior pellet counting experience’ (Table 1; see survey questions sent to observers in S2 Fig in S1 File).

Additionally, we assigned a forest maturity index (cutting class), field-layer composition [47], tree density, and dominant canopy species from a separate dataset, which was collected in 2018 [45]. These covariates should not change within one or two years unless the area was clearcut or thinned, which was indicated in the ‘forestry actions’ variable collected in 2019 and 2020 (Table 1). In this case, clearcut or thinned plots were dropped from the dataset.

### Density estimates

We calculated density separately for each year and for single and double observer counts. We compared counts from single observer surveys (i.e., primary observer counts only) and double observer surveys (i.e., primary and secondary observer counts summed), assuming the counts from double observer surveys were closer to ‘truth’. We did this only for plots where double observer surveys occurred. We estimated moose density (*D*) following standard methods:

$$D=\frac{n}{a*t*d}$$ Eq 1

where *n* is the number of detected pellet groups, *a* is the area sampled, *t* is the accumulation period in days (usually based on time elapsed since first snow fall), and *d* is the daily defecation rate [21, 27–30]. We assumed an average defecation rate of 14 pellet groups per day for moose [21, 29] and an accumulation period of 183 days (30 October –30 April). We calculated density ranges based on minimum and maximum defecation rates (min. 13 and max. 23 moose pellet groups per day; [48–50]). We defined the effective sampling area as the number of plots multiplied by the plot area (100 m^2^). We calculated density by dividing abundance by the effective sampling area.

### Multinomial-Poisson mixture models

We estimated detection probabilities from double observer survey data using multinomial-Poisson mixture models [51, 52]. We used a multinomial distribution for the observation state (i.e., the observed counts), and a Poisson distribution became the latent (i.e., unobserved) variable. We used a structure for dependent double observer surveys by specifying multinomial cell probabilities according to (i) the probability observer 1 but not observer 2 detected the pellet group (column 1; Fig 3); (ii) the probability that observer 2 but not observer 1 detected the pellet group (column 2); and (iii) the probability that both detected the pellet group (column 3). We iteratively included a single covariate on detection probability (*p;* all covariates described in Table 1) and cutting class as a single covariate on abundance [53]. We also specified a null model (~1 on *p*, ~1 on abundance), resulting in 12 candidate models. We used Akaike’s Information Criterion corrected for small sample sizes (AICc) and model weights (w_i_) for model selection. We considered models <2 ΔAIC to be equally supported and chose the simpler model as the ‘best’ model [54]. We ran models using package ‘unmarked’ [55] in program R [56].

## Results

### Pellet counts

**Fig 4:**
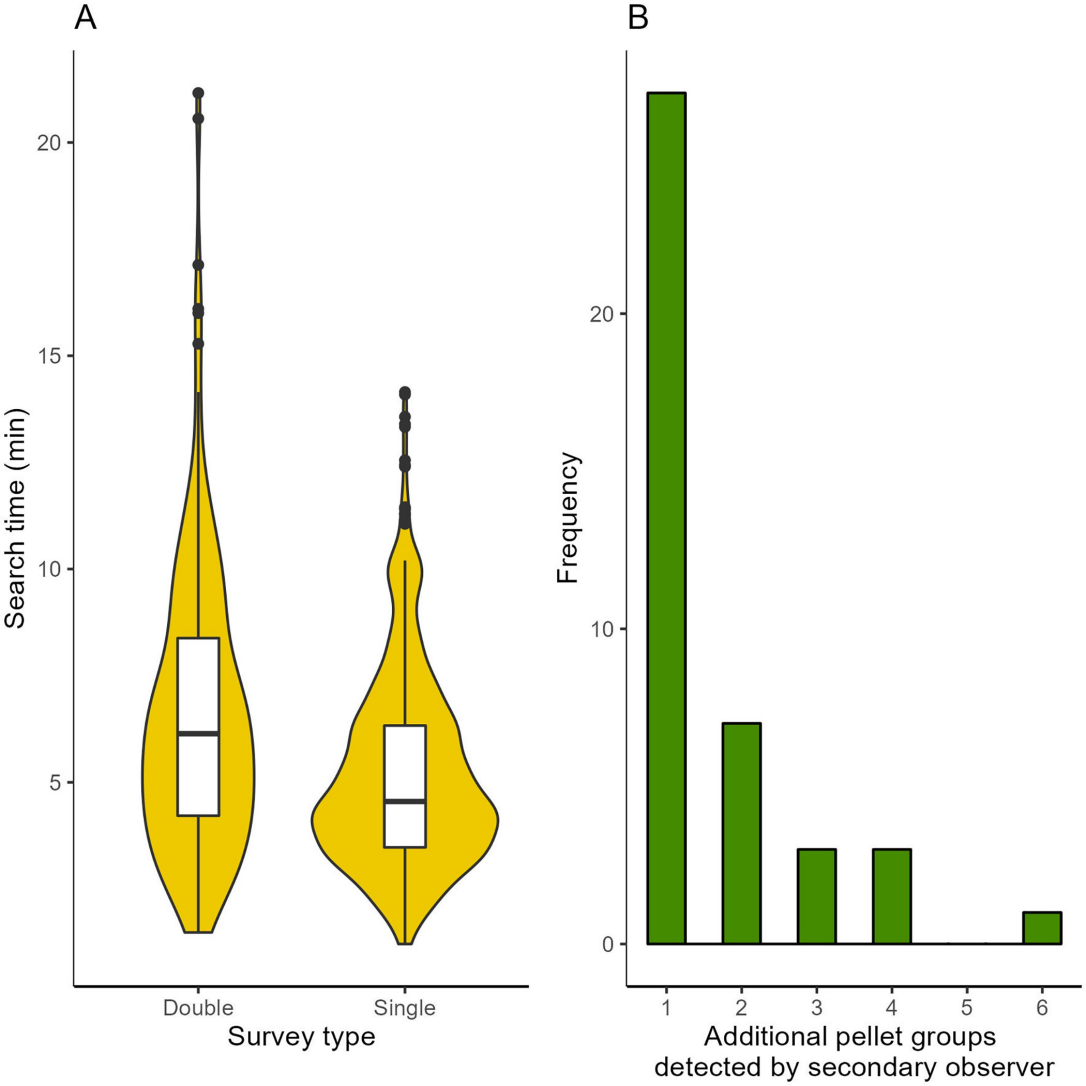
A) Box and violin plot of search time by survey type; B) distribution of additional pellet groups detected by the secondary observer. Surveys were conducted in Norway 2019–2020.

**Fig 5:**
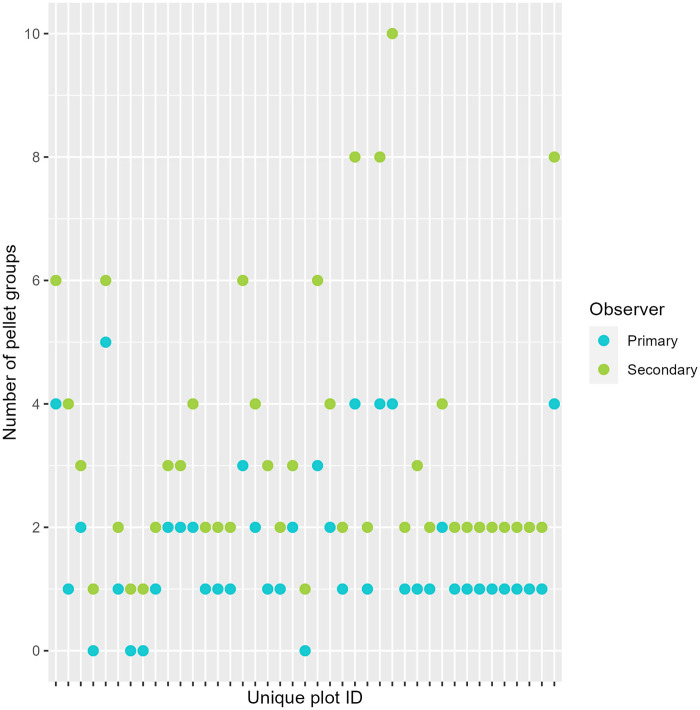
The differences in moose pellet group counts between primary and secondary observers for unique plots. Here, we visualized data only where counts between primary and secondary observers differed (82 plots). Data were subset from the full dataset (325 plots) and were collected in Norway (2019–2020).

We visited 192 and 145 plots in 2019 and 2020, respectively. Seven plots were clearcut and five had missing data from 2018 so we could not reliably assign tree density, field layer, and dominant tree species and dropped these plots from the analysis (new sample size = 325). Across years, 88% of double observer surveys occurred at two of the three sites (Gravberget: n = 161; Ljørdalen: n = 123; Plassen: n = 41). Of the 325 plots, we counted 230 moose pellet groups with a mean count of 0.68 pellet groups per plot (SD = 1.55). We did not detect pellet groups in 70% (n = 229) of the plots. Pellet counts were highest among dwarf shrub (mean pellet counts per plot = 0.52, SD = 1.24) and lichen (mean pellet counts per plot = 0.53, SD = 0.99) field layer types. Fifty percent (n = 13) of our observers had no prior field experience, 31% (n = 8) had 0–6 months experience, 19% (n = 5) had >7 months of experience. Single observers spent less time surveying than double observers (Fig 4A). The secondary observer detected additional pellet groups (mean number of pellet groups detected by secondary observer = 0.20, SD = 0.67) not seen by the first observer (mean number of pellet groups detected by first observer = 0.48, SD = 1.12). This indicated a potential source of detection error. When pellet groups were present (e.g., combined observer count > 0; n = 96) the secondary observer saw additional pellet groups 42% (n = 40) of the time (Fig 4A). When counts by primary and secondary observers did not match, the secondary observer often saw additional pellet groups that went undetected by the primary observer (Fig 5).

### Density estimates

**Fig 6:**
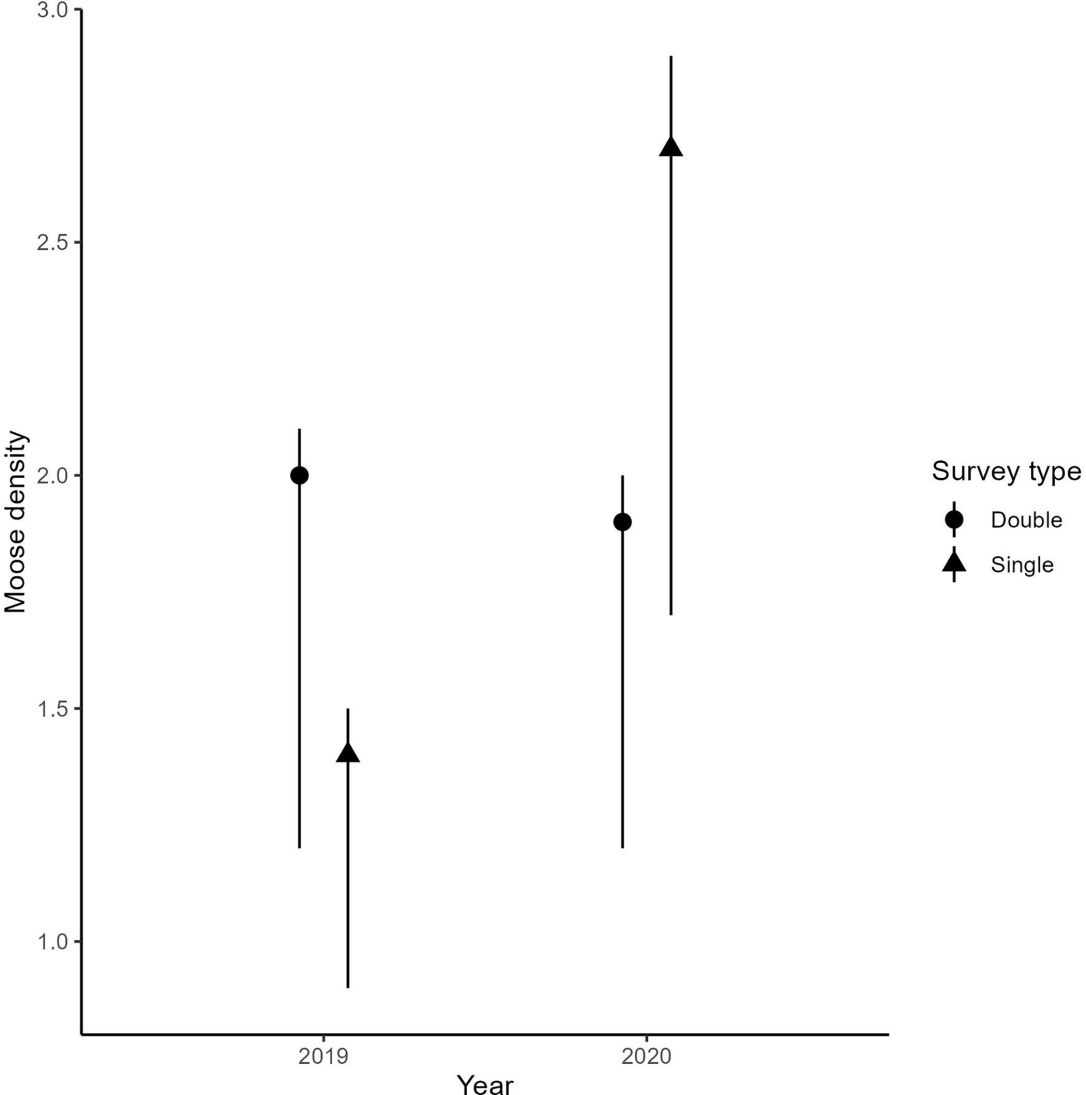
The number of moose per km^2^ (i.e., moose density) by year and survey type (either double or single observer surveys).

We calculated density estimates separately for 2019 and 2020. In 2019, double and single observer density estimates were 2.0 (min–max: 1.2–2.1) and 1.4 (min–max: 0.9–1.5) moose per km^2^, respectively. In 2020, double and single observer density estimates were 2.7 (min–max: 1.7–2.9) and 1.9 (min–max: 1.2–2.0) moose per km^2^, respectively (Fig 6).

### Multinomial-Poisson mixture models

**Fig 7:**
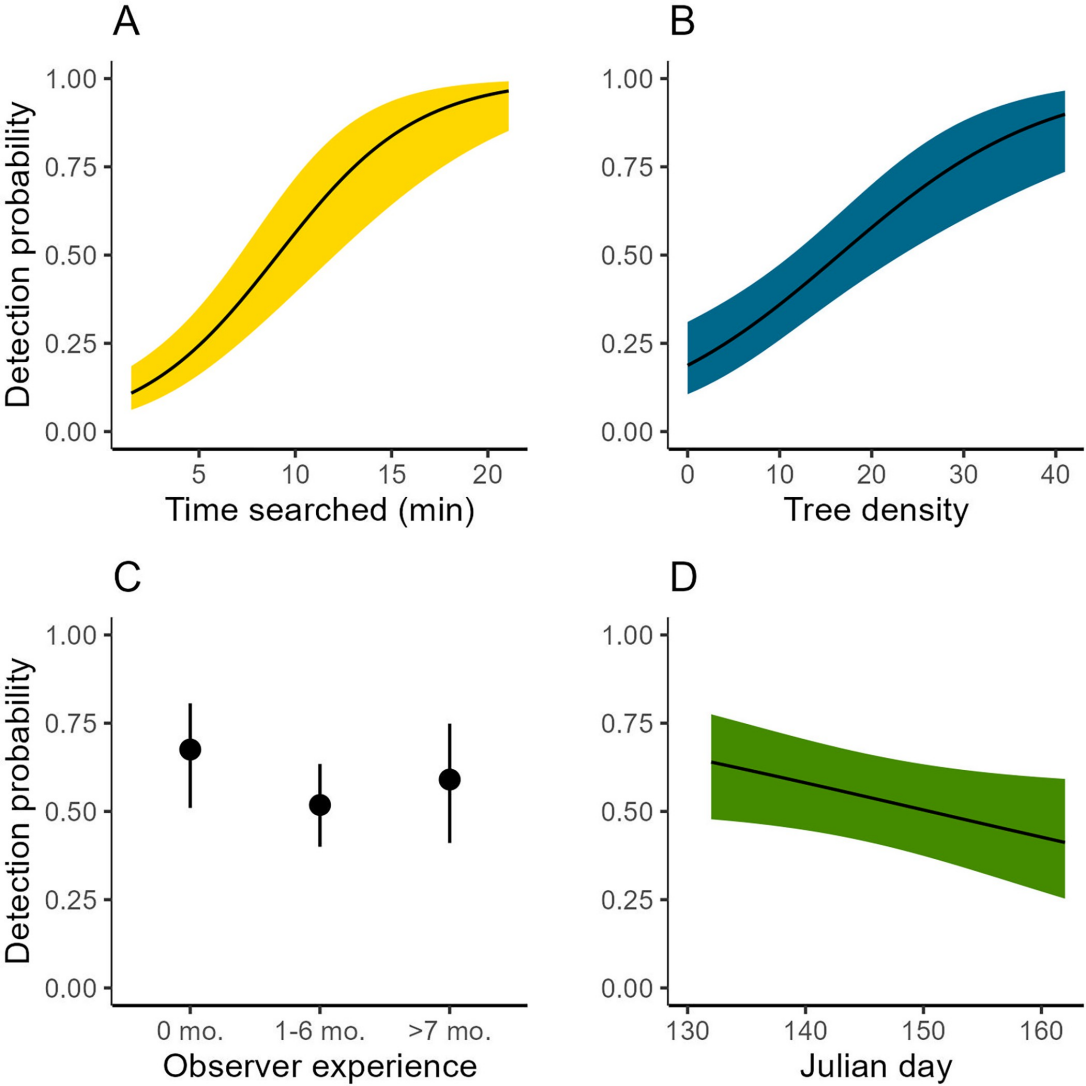
Prediction plots showing detection probabilities for A) the time searched in minutes; B) number of trees taller than 0.30 cm in the plot; C) prior observer experience in field research; D) Julian day that sampling occurred. Ribbons and error bars represent 95% confidence intervals. Predictions were made from models listed in Table 2.

The top-ranked model included time searched (Table 2) where *p* increased with search time (Fig 7A). At 10 minutes search time, detection probability was 0.60 (95% CI = 0.42–0.75). At 20 minutes search time, detection probability was 0.94 (95% CI = 0.81–0.99). Looking at lower-ranked models (Table 2) *p* increased with tree density (Fig 7B), *p* was lowest for moderately experienced observers (Fig 7C), and *p* decreased slightly as Julian day increased (Fig 7D). Interestingly, field layers ranked low (Table 2) in relative parsimony. Detection probabilities were highest in field layers dominated by dwarf shrubs (S3 Fig in S1 File) but this was also the most common field layer type (S4 Fig in S1 File). We back-transformed estimates of *p* from the null model: *p* = 0.56 (SE = 0.06) and abundance = 0.84 (SE = 0.08). This suggests that when a pellet group was present, it went undetected 44% of the time by the first observer. The only covariate used for abundance was cutting class. Most moose pellet groups were in cutting class two, i.e. young forest stands (S5 Fig in S1 File).

**Table 2:** Model selection table from multinomial Poisson mixture models. Covariates listed here were used only on detection probability (p). For all models, cutting class was the sole covariate used on abundance and is not listed in the table.

Model	K	AICc	ΔAICc	w_i_	LL
Time searched	8	948.65	0.00	1.00	-466.10
Tree density	8	963.48	14.83	0.00	-473.51
Julian day	8	978.07	29.42	0.00	-480.81
Observer experience	9	980.18	31.53	0.00	-480.80
Snow cover	8	980.99	32.34	0.00	-482.27
Dominant tree	10	981.42	32.77	0.00	-480.36
Residual cover	8	982.00	33.35	0.00	-482.77
Time of day	8	982.71	34.05	0.00	-483.13
Field layer	12	983.69	35.04	0.00	-479.35
Weather	12	983.75	35.10	0.00	-479.38
Pellet counting experience	9	983.89	35.24	0.00	-482.66
Null model	2	1077.19	128.54	0.00	-536.58

## Discussion

Pellet counts are a commonly used method to monitor ungulates because they are relatively cheap, it is easy to train inexperienced workers, and do not require expensive field equipment. Yet, counts are rarely without error [1, 57] and there are known issues with pellet counts, such as uncertainties with pellet decay and incomplete observations [58]. This paper addressed the latter where we completed 16% of annual plot surveys as double observer surveys to quantify and assess detection error. We found more pellet groups were detected during double observer surveys, and modelling indicated we detected 56% of pellet groups when they were present. While our estimates indicate we missed pellets, and may be cause for concern, they were similar to estimates of *p* in other deer studies. For example, in one study, teams of inexperienced observers detected on average 68% of the deer (fallow deer [*Dama dama*], roe deer, white-tailed deer [*Odocoileus virginianus*]) pellet groups detected by professional biologists (i.e., experienced observers; Buesching et al. 2014), meaning inexperienced observers missed 32% of the known pellet groups. The consequences of missing pellet groups could be small if the spatial and temporal scale of the estimates are also small, as was the case in our study.

In Scandinavia, hunting is a primary driver of moose mortality [59], where between 18 and 35% of the population is harvested each autumn [31, 32]. Pellet counts are used by both researchers and managers as indices of absolute and relative abundance, density, or population trend [21, 27–30]. For a research example, Zimmermann et al. [21] used pellet counts to estimate the relative density of moose and other deer species to understand functional responses of wolves to their prey. For a management example, in central Sweden (Svealand), pellet counts are used to supplement observed moose (‘sett elg’) to help set moose harvest quotas [60], though the use of pellet counts may decline in the future (F. Widemo, pers. comm.).

The scale of our study was small relative to moose distributions in Scandinavia (Fig 1, and one could argue that our sites were too small (minimum site area = 38.68 km^2^; maximum site area = 55.57 km^2^) to estimate moose density, relative to an average moose winter home range (across Sweden 10.81 km^2^; SD = 6.55; [61]; specific to our study region in Norway 42.15 km^2^; SE = 8.55; [62]). Yet, if we think of our density estimates as simply a mathematical exercise, and we assume our double observer counts represent “true” values, the moose density would be 1.4 times larger than if we had used single observer counts only. This result highlights a possible mismatch between on-the-ground moose densities and management objectives. For example, low moose harvest rates could lead to unwanted population growth and increased browsing damage for commercial forestry.

In addition to understanding how systematic biases could influence density estimates, detection probability covariates provided insights into our sampling biases. First, detection probabilities were highest for the least and most experienced observers. While this is not an intuitive result, it is supported by findings from other studies where inexperienced observers performed better than experienced observers. For example, technicians new to the sampling protocol in a non-invasive genetic sampling study in the USA collected more scats, were more accurate in identifying the target species, and collected more high-quality samples for genetic amplification relative to technicians already familiar with the protocol [63]. The authors attributed these findings to inexperienced observers being choosier, meaning they likely sampled from higher-quality scats, and experienced observers possibly became bored or fatigued. This could certainly be the case in our study, where the same field protocols are completed multiple times per day. Interestingly, observers with moderate experience (7–12 months prior experience) in our study searched the longest but had the lowest detection probability.

Second, we also found that detection probability increased with increasing tree density. Given this counterintuitive result, we suspected this was a result of increased search time in plots with higher tree density. Our data show an increase in time spent searching the plot until a tree density of 15 trees per plot, after which search time stabilized around seven minutes (S6 Fig in S1 File). High tree densities could also reduce field layer complexity due to canopy shading. Likely there are interactions present between tree densities and search time, which we did not test in this paper. Third, we found that *p* decreased with Julian day; an increase by one week (7 days) decreased *p* by 0.9. This makes sense as green-up of the field layer can conceal pellets, as confirmed by previous studies [24]. Fourth, differences in detection probabilities based on field layer types indicate potential habitat biases. However, our field study was dominated by dwarf shrub field layer (70% of plots) so the lack of variation prevents strong concerns about habitat biases.

How can pellet count methods be improved? We provide three recommendations. First, we recommend that projects relying on pellet counts do a subset of plots as double observer surveys to identify sources of error or bias. While we implemented a dependent double observer method because it has higher precision and is more time efficient in the field than independent double observer methods [13], projects could modify our methods to do independent double counts. For example, the Scandinavian Wolf Research Project (SKANDULV; e.g. [21, 64]) conducts deer pellet counts by surveying the plot in a similar manner as described in Fig 3. However, they have a single observer walk the inner and outer circles twice, walking each circle first clockwise and then counter-clockwise (i.e., they make four ‘rounds’). This is an intensive searching method that could be easily changed to estimate detection rates: the first walk around could be recorded as a ‘first count’ and the second walk would be the ‘second count’. Additionally, a more experienced observer (i.e., local people with extensive tracking experience) could be paired with less experienced observer to increase learning and detection probabilities.

A second recommendation for improving pellet counts is that once sources of error are identified, they can be accounted for in the field and in modeling. In our case, search time was an important variable for detection. In the field, minimum search times could be implemented based on desired detection probabilities, and ‘time searched’ should be recorded. At the office, ‘time searched’ could be included in models and future single-observer counts could be adjusted based on the known detection probabilities. Third, we recommend doing a subset of single observer plots as double observer surveys for several years. The data collected for this study only represented two years. In this time, we relied on large groups (> 16 observers) of inexperienced observers. However, every third year (e.g., 2018, 2021) we have smaller groups (4–8 observers) who spend up to two months in the field. Observers thus get more experience within the field season, versus only a few days where observers have only a short ‘learning window.’ We might see even stronger trends in *p* based on prior observer experience.

The future of population monitoring is changing. Advances in non-invasive genetic techniques have made individual assignment of moose from pellets possible [65]. While more costly for the same spatial extent, this may represent a possibility for future moose population monitoring, as is the case for other wide-ranging mammals [66, 67]. One of the take-homes from this study is that large uncertainties exist in management decisions and being able to identify sources of error can help reduce those uncertainties. For large-scale efforts like those in Svealand, Sweden, pellet counts are completed mostly by volunteer hunters (‘citizen scientists’). This represents an excellent database, but the numerous observers with differing prior field experience levels likely introduces additional variation into the observed pellet groups. We maintain that pellet counting is an important tool for monitoring, as it is an easy method to implement across large areas. However, our results highlight the uncertainties with a standard method and recommend research and management projects complete a subset of plots as double observer surveys to identify and quantify uncertainties.

## Supporting information

S1 File (DOCX)
