# Supplementary material for: The importance of evaluating standard monitoring methods: Observer bias and detection probabilities for moose pellet group surveys
Source: PLoS One. 2022 Jul 27;17(7):e0268710. doi: 10.1371/journal.pone.0268710 (PMC9328498; doi:10.1371/journal.pone.0268710)
Supplement: S1 File — (DOCX) [file pone.0268710.s001.docx]

Supplementary information for manuscript titled: The importance of evaluating standard monitoring methods: Observer bias and detection probabilities for moose pellet group surveys

**Authors**:

Anne E. Loosen (orcid.org/0000-0002-6145-4044)

Olivier Devineau (orcid.org/0000-0002-7625-2816)

Barbara Zimmermann (orcid.org/0000-0001-5133-9379)

Karen Marie Mathisen (orcid.org/0000-0001-5166-8009)

**Submitted to PLOS ONE**


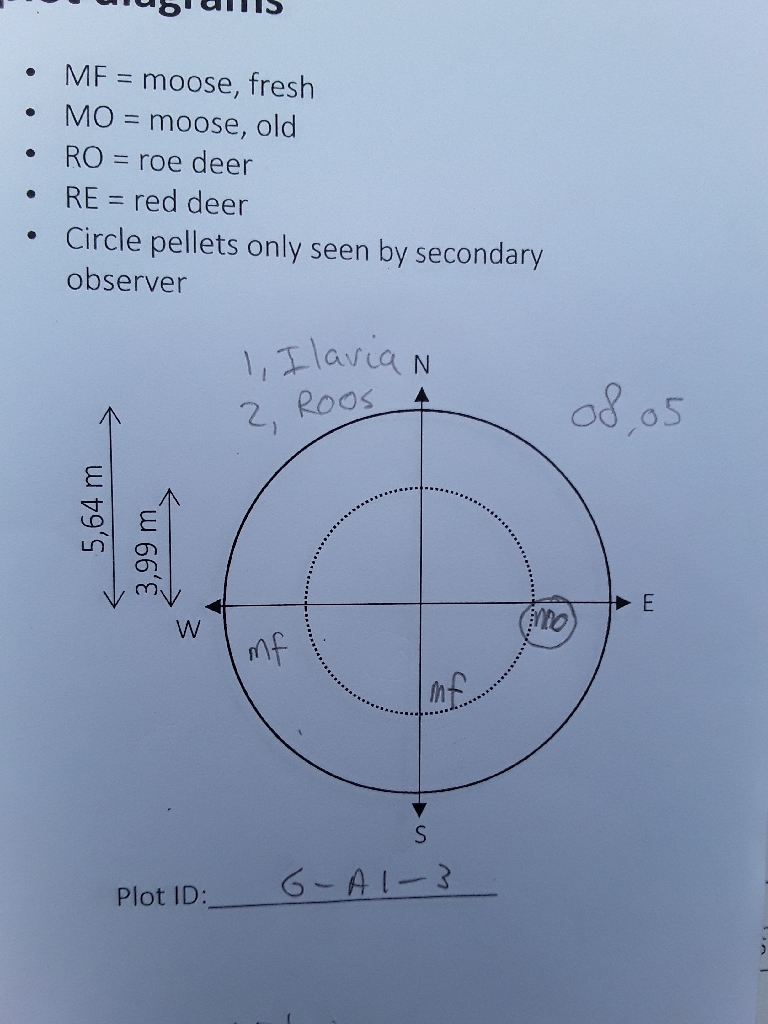


Figure S1. Example of a double observer diagram completed in the field. Observers used a rope, which was marked at 5.64 m (solid black circle) and 3.99 m (dotted circle), to measure the plot radii. Observers walked in the inner circle first, zig-zagging within the circle to ensure the area was properly surveyed. Once the smaller circle was surveyed, observers moved to the outer circle and searched in the opposite direction to ensure pellet groups obscured by vegetation could be seen. Cardinal directions are indicated to help visually break the plot into sections. Abbreviations for mf, for example, indicate fresh moose pellets and their location within the circular plot.


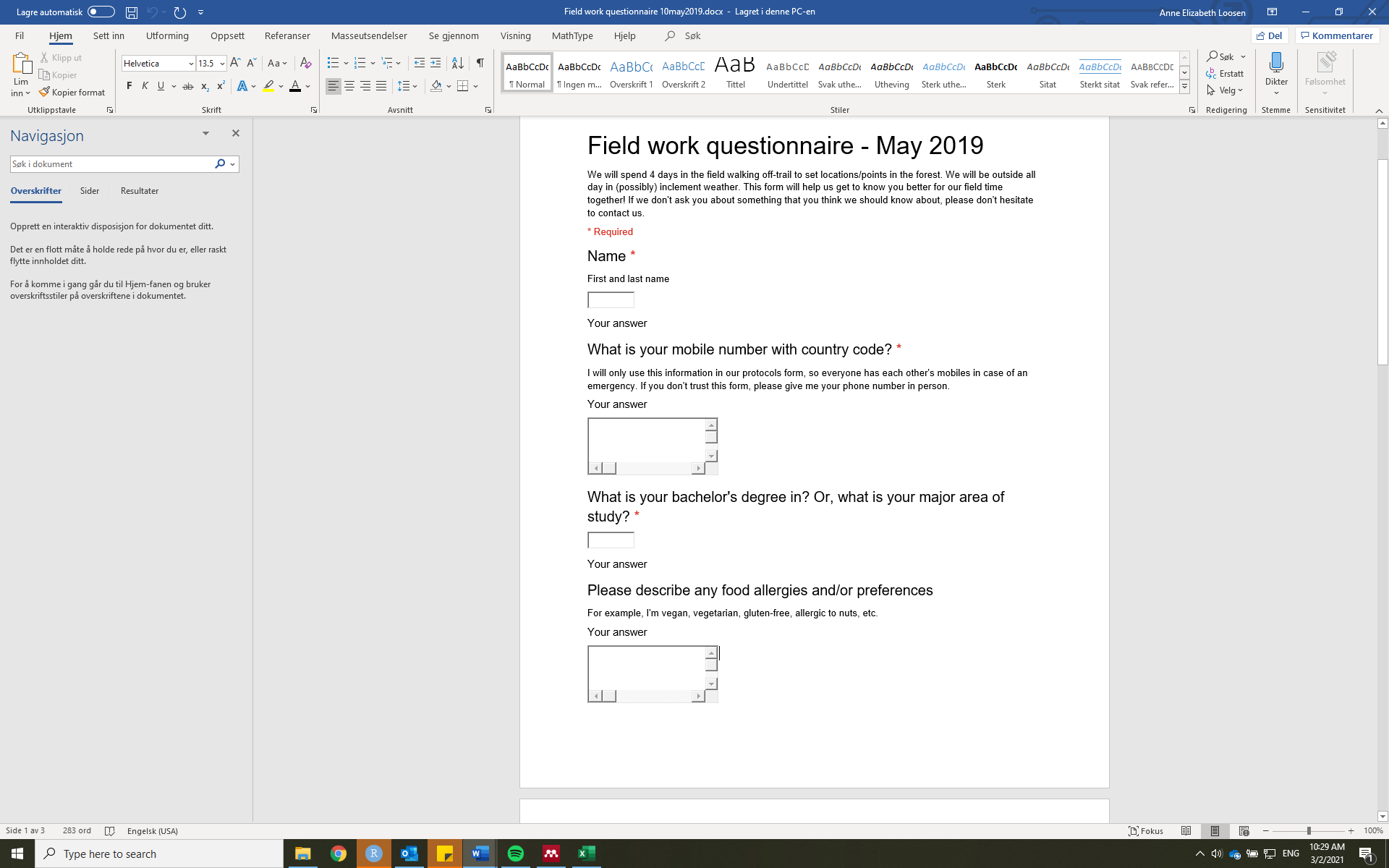

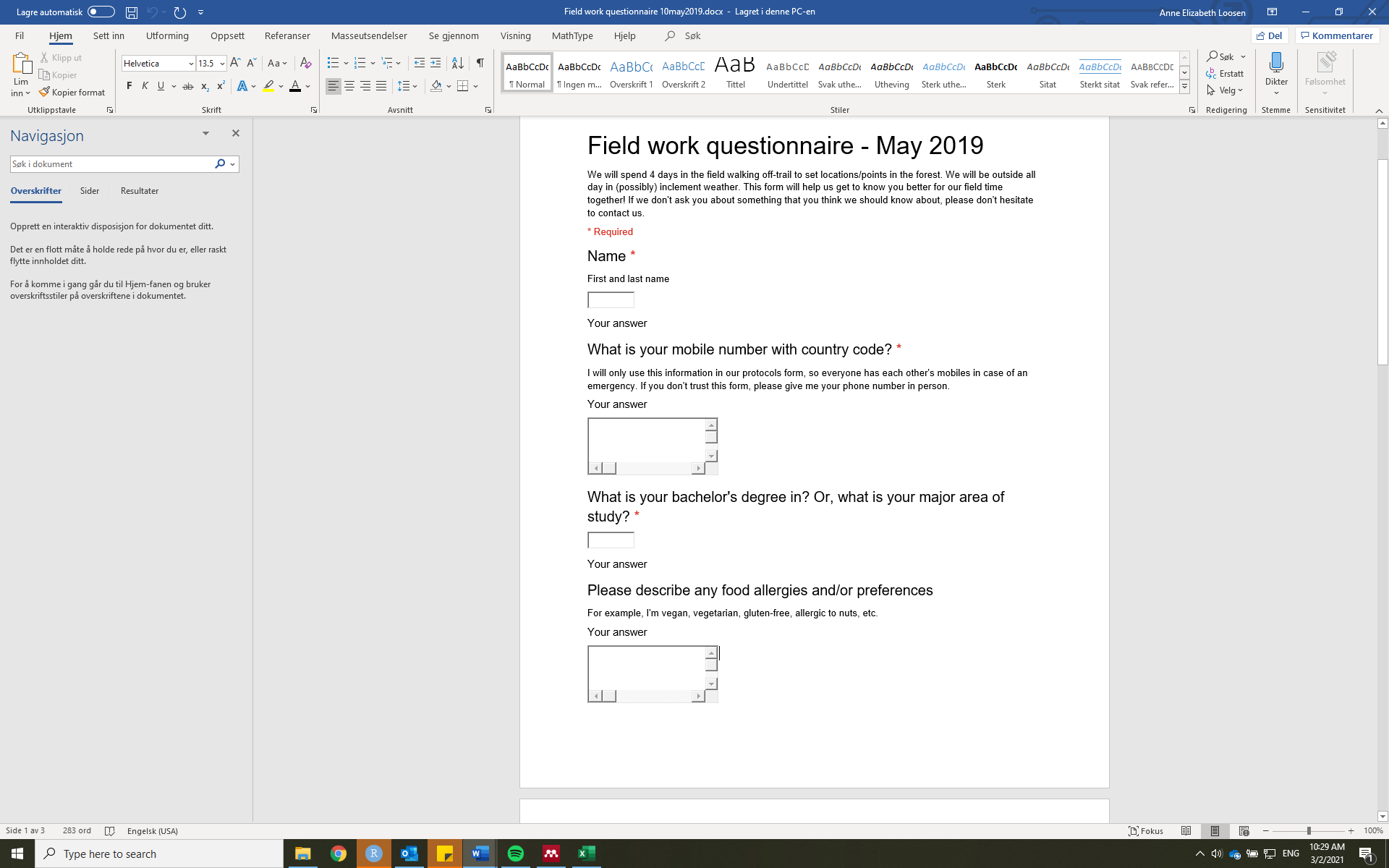


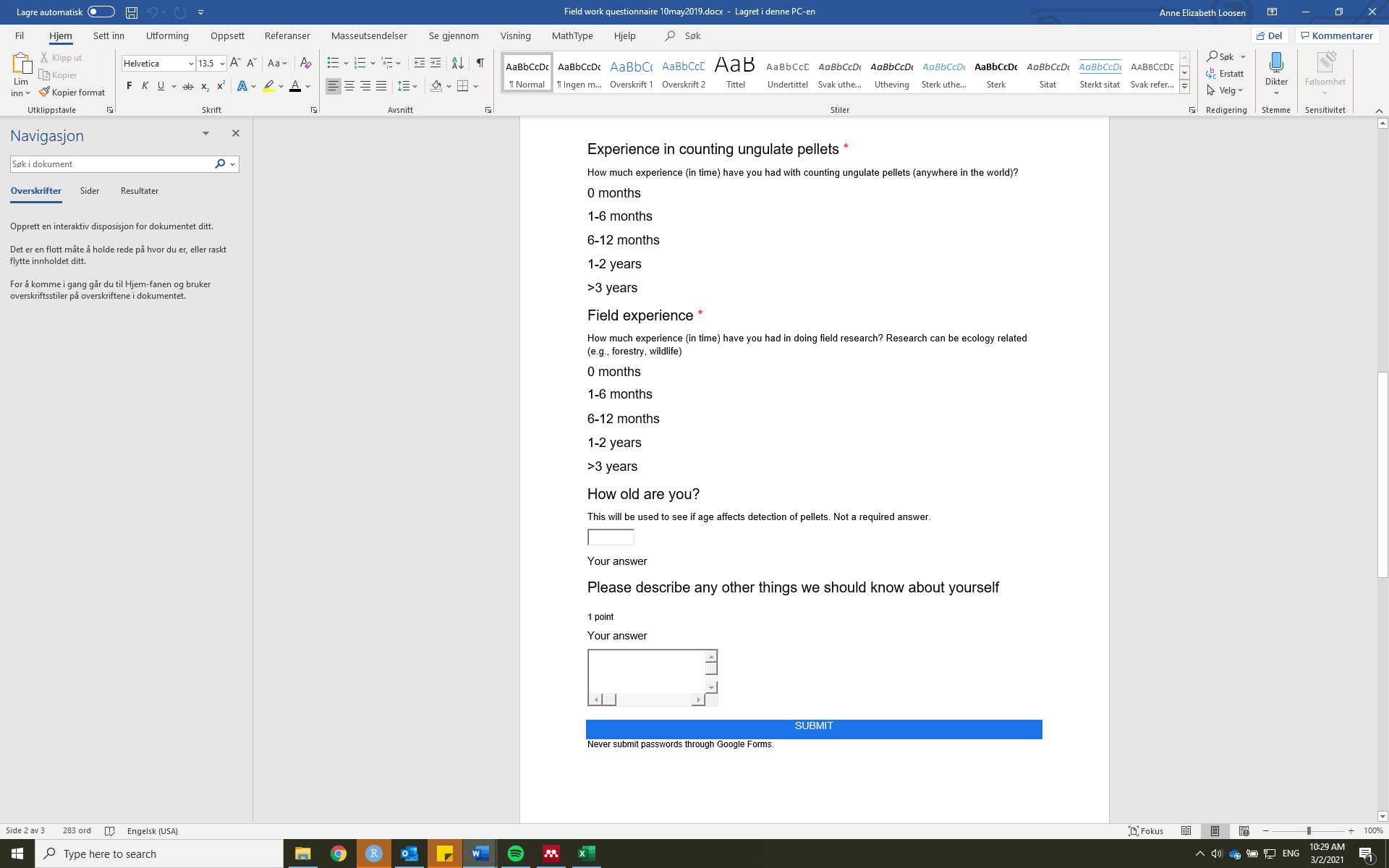


Figure S2. Survey questions completed by each observer.


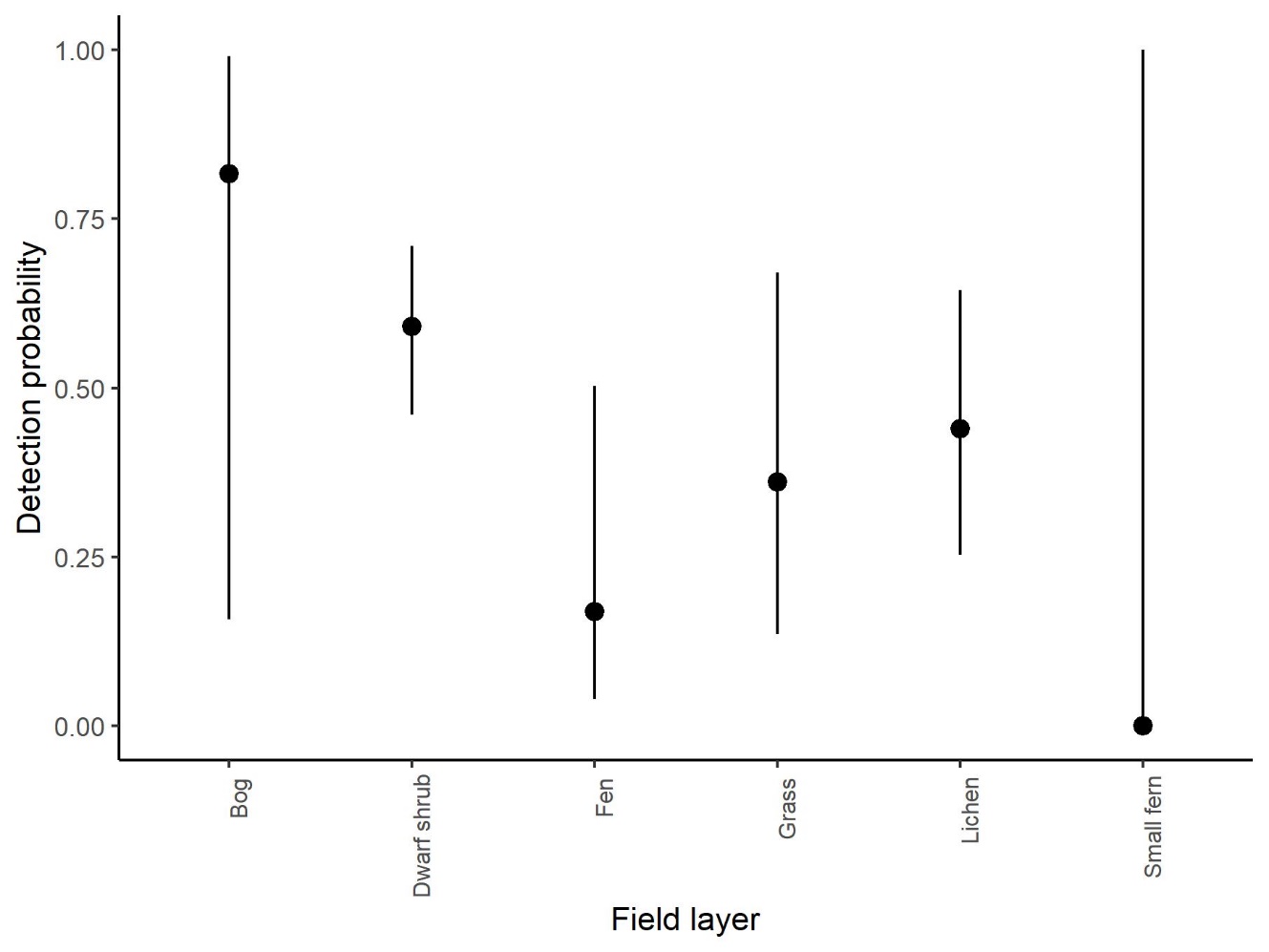


Figure S3. Detection probabilities by field layer type. Error bars represent 95% confidence intervals. Data were from Norway 2019–2020.


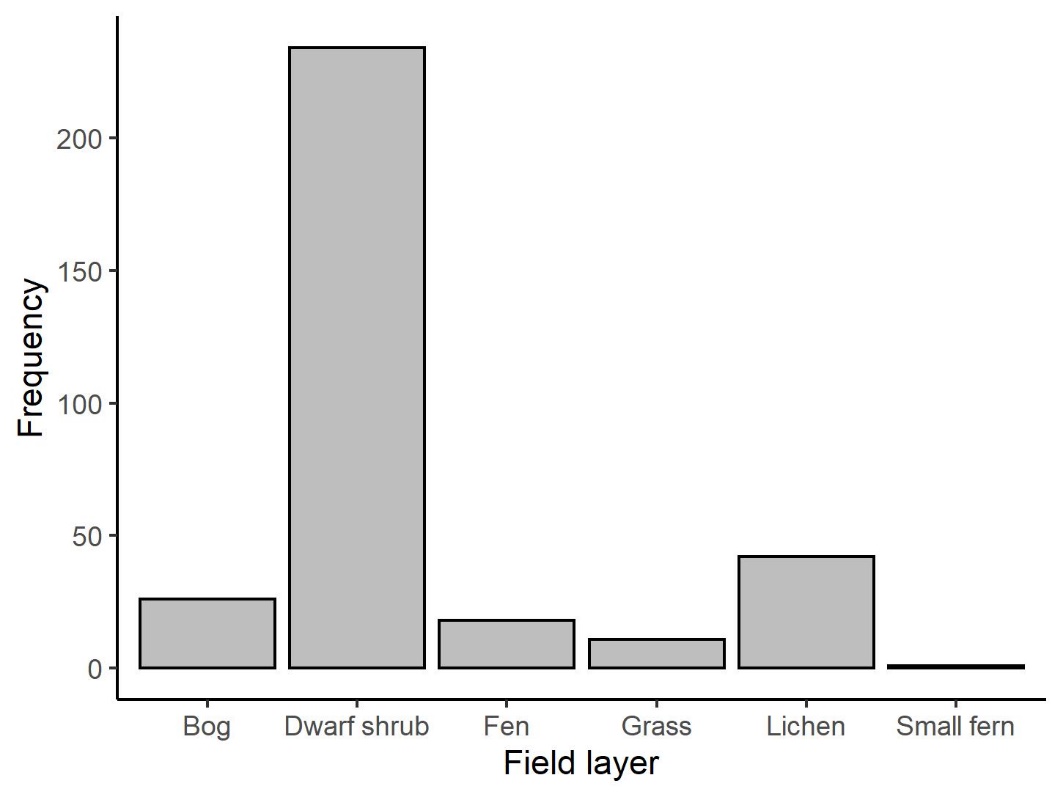


Figure S4. The distribution of field layer classifications in our data. Data were from Norway 2019–2020.


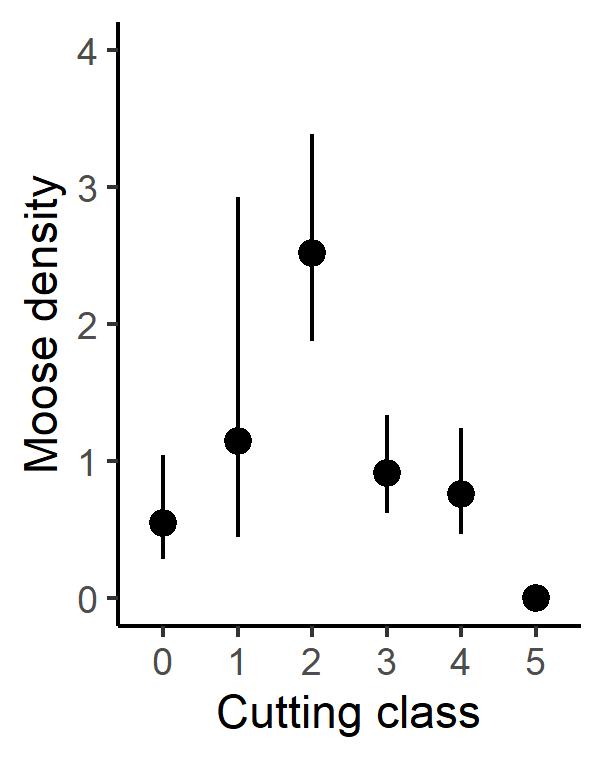


Figure S5. Predicted moose density (number of moose per km^2^) by cutting class, which are graphed in order of maturity (1 = clearcut, 5 = old-growth forest; see Table 1 for the variable description). Predictions were made from top-ranked multinomial-Poisson mixture model. Error bars represent 95% confidence intervals.


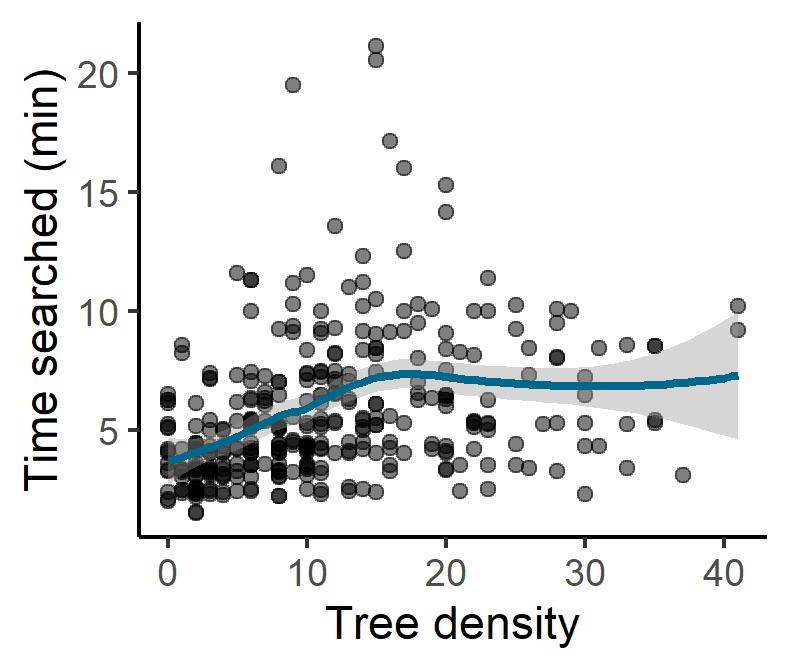


Figure S6. Scatter plot and loess smoother (green line) of tree density and time spent searching the plot. Tree density was number of trees taller than 0.30 cm per plot. Grey ribbon represents 95% confidence interval. Data were from Norway 2019–2020.
